# Association between dietary vitamin C intake and gout among American adults

**DOI:** 10.3389/fimmu.2024.1431323

**Published:** 2024-09-13

**Authors:** Yadan Zou, Yongyu Liu, Shengguang Li

**Affiliations:** ^1^ Department of Rheumatology and Immunology, Peking University International Hospital, Peking University, Beijing, China; ^2^ Department of Rheumatology and Immunology, Peking University People’s Hospital, Peking University, Beijing, China

**Keywords:** vitamin C, gout, inverse association, dose-response, a large population study

## Abstract

**Introduction:**

Gout is a common type of inflammatory arthritis. Vitamin C is a potent antioxidant that neutralizes reactive oxygen species. However, the association between dietary vitamin C levels and gout remains unclear. This study evaluated the relationship between dietary vitamin C intake and gout.

**Methods:**

Cross-sectional data from individuals aged > 20 years who participated in the National Health and Nutrition Examination Survey between 2013 and 2018 were collected. Details on gout, dietary vitamin C intake, and several other essential variables were recorded.

**Results:**

There were 12589 participants, 5% (652/12589) of whom experienced gout. Compared with individuals with lower vitamin C consumption in the Q1 group (≤19.9 mg/day), the adjusted odds ratio(OR)values for dietary vitamin C intake and gout in the Q2 group (19.9–49.7 mg/day), Q3 group (49.7–110.375 mg/day), and Q4 group (≥110.375 mg/day) were 0.87 (95% confidence interval (CI): 0.69–1.1, *P* = 0.237), 0.81 (95% CI: 0.64–1.02, *P* = 0.076), and 0.77 (95% CI: 0.6–0.99, *P*= 0.042), respectively. Accordingly, the association between dietary vitamin C intake and gout exhibited an L-shaped curve (nonlinear, *P* = 0.245) in a restricted cubic spline. Subgroup analysis revealed significant interactions between vitamin C levels and gout according to sex (*P* < 0.05). When we used data on dietary vitamin C from the second survey, we observed a similar inverse association between vitamin C intake and gout. The vitamin C was also negatively associated with hyperuricemia (OR, 0.94; 95% CI, 0.9–0.98, *P*=0.005). Compared with Q1, the adjusted OR values for dietary vitamin C and hyperuricemia in Q2, Q3, and Q4 were 0.77 (95% CI: 0.69–0.86, *P* = 0.65), 0.81 (95% CI: 0.72–0.91, *P* = 0.014), and 0.72 (95% CI: 0.64–0.81, *P* < 0.001), respectively. No association was observed between vitamin C supplementation and gout.

**Conclusion:**

The population-based data indicate that dietary vitamin C intake is inversely associated with gout. These findings support the potential role of vitamin C in preventing gout.

## 1 Introduction

Gout is a prevalent form of inflammatory arthritis, characterized by the deposition of monosodium urate crystals in the joints and tissues, affecting over 5% of males in the United States and leading to increasing hospitalization rates in the US and Canada (1). Gout presents recurrent excruciating flares, increasing the risk for heart attacks (2), strokes, and debilitating arthritis if left untreated (3). The etiology of gout is multifaceted, involving genetics, diet, and lifestyle. Recent studies (4–6) have explored the potential role of micronutrients such as vitamin C in managing and preventing hyperuricemia and subsequent gout flares.

Vitamin C (ascorbic acid) is a potent water-soluble antioxidant known to scavenge free radicals and reduce oxidative stress. It is implicated in the onset and exacerbation of inflammatory diseases, including gout. The antioxidant properties of vitamin C may help to mitigate the oxidative breakdown of purines in the body, a metabolic process that significantly contributes to the total uric acid pool. By potentially reducing the rate of purine metabolism, vitamin C can indirectly reduce uric acid production. Molecular research suggests vitamin C can dissolve urate crystals (7) and has antioxidant functions.

Moreover, vitamin C can influence the activity of urate transporters such as urate transporter 1(URAT1) and sodium-dependent anion cotransporters, which are involved in the reabsorption of uric acid in the proximal tubules of the kidneys (8, 9). Third, increased consumption of vitamin C may potentially enhance kidney function and increase the glomerular filtration rate (4), thereby enhancing urate excretion in the urine (10). Both human and animal studies indicate that administering vitamin C boosts renal plasma flow and glomerular filtration rate while mitigating increases in arterial pressure (11). Finally, vitamin C inhibits urate-induced inflammation by acting as an antioxidant. It can inhibit the activity of the thioredoxin-interacting protein (TXNIP) in nuclear factor kappa B (NF-κB) signaling, which is involved in the activation of the NLR family pyrin domain containing 3 inflammasomes (NLR*P*3), a key mediator of urate-induced inflammation (12). These mechanisms contributed to the overall reduction in uric acid levels observed following vitamin C supplementation.

Currently, evidence regarding the association between dietary vitamin C levels and gout is insufficient. Given the potential link between dietary vitamin C and gout, we hypothesized that dietary vitamin C exerts a protective effect against gout development. Therefore, this study aimed to investigate the impact of changes in dietary vitamin C levels on gout in a large population.

## 2 Materials and methods

### 2.1 Study population and data collection

This cross-sectional study used NHANES data from 2013–2018, administered by the Centers for Disease Control and Prevention (CDC). The NHANES aims to assess the health and nutritional status of non-institutionalized Americans using a stratified multistage probability survey (13). Data collection included demographic details, health assessments, and laboratory tests via a mobile examination center (MEC) with ethical approval from the National Center for Health Statistics (NCHS) Ethics Review Committee. Written informed consent was obtained from all the participants. The NHANES data was accessed from their website (http://www.cdc.gov/nchs/nhanes.htm) (accessed March 1, 2022). Our study included individuals aged > 20 years who completed the interviews. Pregnant females or those with missing data on gout, dietary vitamin C intake, or covariates were excluded.

### 2.2 Vitamin C intake measurement and outcomes

Vitamin C intake from the first survey in the NHANES database is measured through a 24-hour dietary recall conducted during the participant’s initial visit to the MEC. In this face-to-face interview, trained dietary interviewers used the automated multiple-pass method (AMPM) to collect detailed information about all foods and beverages consumed by the participants in the previous 24 hours. Nutrient content, including vitamin C, was calculated using the food composition database. These data represent participants’ vitamin C intake from food and beverages during the first survey.

Vitamin C intake from the second survey was measured through a second 24-hour dietary recall, usually conducted via telephone, a few days after the initial interview. The process mirrored the first survey, with interviewers using the AMPM again to collect detailed dietary information about the participants’ consumption over the previous 24 hours. Vitamin C content was calculated using the same food composition database, measuring vitamin C intake from a different time point. This helped account for day-to-day variability in the participant’s diet. Vitamin C comprises dietary vitamin C, and supplemental Vitamin C. Subjects were categorized into four groups according to dietary vitamin C intake.

During the home interviews, all participants were asked, “Has a doctor or other health professional ever informed you that you had gout?” Participants who answered “yes” were defined as gout. Serum uric acid (SUA) concentration was measured at the NHANES Laboratory for all three cycles. Hyperuricemia was defined as an SUA concentration >6mg/dL (14).

### 2.3 Covariates

A range of potential covariates were evaluated based on the existing literature (15–17), encompassing age, sex, marital status, race/ethnicity, education level, family income, smoking habits, physical activity levels, hypertension, diabetes, coronary heart disease, alcohol consumption, and dietary supplement usage. Race/ethnicity was stratified into non-Hispanic White, non-Hispanic Black, Mexican American, and other ethnic groups. Marital status was categorized as married, cohabiting, or living alone. Education level was divided into less than 9 years, 9–12 years, and more than 12 years, following the guidelines of a US government report. Family income was classified into three groups based on the poverty income ratio (PIR): low (PIR ≤ 1.3), medium (PIR > 1.3 to 3.5), and high (PIR > 3.5). Smoking status was defined as never smoker (smoked less than 100 cigarettes), current smokers, or former smokers (ceased smoking after consuming > 100 cigarettes), in line with established definitions from previous research. Physical activity levels were categorized as sedentary, moderate (at least 10 min of light activity resulting in mild-to-moderate sweating or increased breathing/heart rate within the last 30 days), and vigorous (at least 10 min of activity resulting in profuse sweating or increased breathing/heart rate within the last 30 days). A history of hypertension, diabetes, and coronary heart disease was determined based on self-reported physician-diagnosed conditions. Alcohol drinking status was determined using the survey question “In any 1 year, have you had at least 12 drinks of any type of alcoholic beverage?” Participants who answered “yes” were classified as alcohol drinkers. Dietary supplement usage was ascertained by querying participants about the nutritional supplements and medications taken in the past month.

### 2.4 Statistical analysis

This study constituted a secondary analysis of publicly available datasets. Categorical variables were presented as percentages (%), while continuous variables are summarized using the mean (standard deviation, SD) or median (interquartile range, IQR), as applicable. Group differences were assessed using one-way analysis of variance (for normally distributed data), the Kruskal–Wallis test (for skewed distributions), and the chi-square test (for categorical variables). Logistic regression models were used to ascertain the odds ratios (ORs) and corresponding 95% confidence intervals (CIs) to examine the association between dietary vitamin C intake and gout. Model 1 was adjusted for sociodemographic factors, including age, sex, race/ethnicity, marital status, education level, and family income. Model 2 is adjusted for complications. Model 3 was fully adjusted and incorporated sociodemographic characteristics, smoking status, physical activity, hypertension, diabetes, coronary heart disease, alcohol consumption, and dietary supplement use.

Additionally, restricted cubic spline (RCS) regression was conducted using four knots at the 5th, 35th, 65th, and 95th percentiles of dietary vitamin C consumption. This analysis aimed to evaluate the linearity and explore the dose-response relationship between dietary vitamin C consumption and gout. The RCS model was adjusted for the covariates included in the logistic regression models to ensure consistency in the analysis.

Moreover, potential effect modifications on the association between dietary vitamin C and gout were examined, encompassing the following factors: sex, age (20–65 years vs. >65 years), family income (low vs. medium or high), hypertension (yes vs. no), coronary heart disease (yes vs. no), and alcohol consumption (yes vs. no). Subgroup heterogeneity was assessed using multivariate logistic regression, and the interactions between subgroups and dietary vitamin C intake were investigated using likelihood ratio testing.

Several sensitivity analyses were conducted to verify the robustness of the findings. First, we conducted a sensitivity analysis that excluded participants with extreme vitamin C intake (>1000 kcal per day). Second, In NHANES, there were multiple surveys related to vitamin C intake, including total intake from the first survey, total intake from the second survey, and intake as a dietary supplement. We further explored the association between vitamin C (based on the second survey) and gout as well as the association between vitamin C (based on dietary supplements) and gout. Third, owing to the close association between gout and hyperuricemia, we explored the relationship between vitamin C levels and hyperuricemia.

We supplemented our analysis by reporting the likelihood ratios for each regression model and conducted a detailed comparison of the models based on the AIC, BIC, and log-likelihood metrics.

## 3 Results

### 3.1 Study characteristics

**Figure 1:**
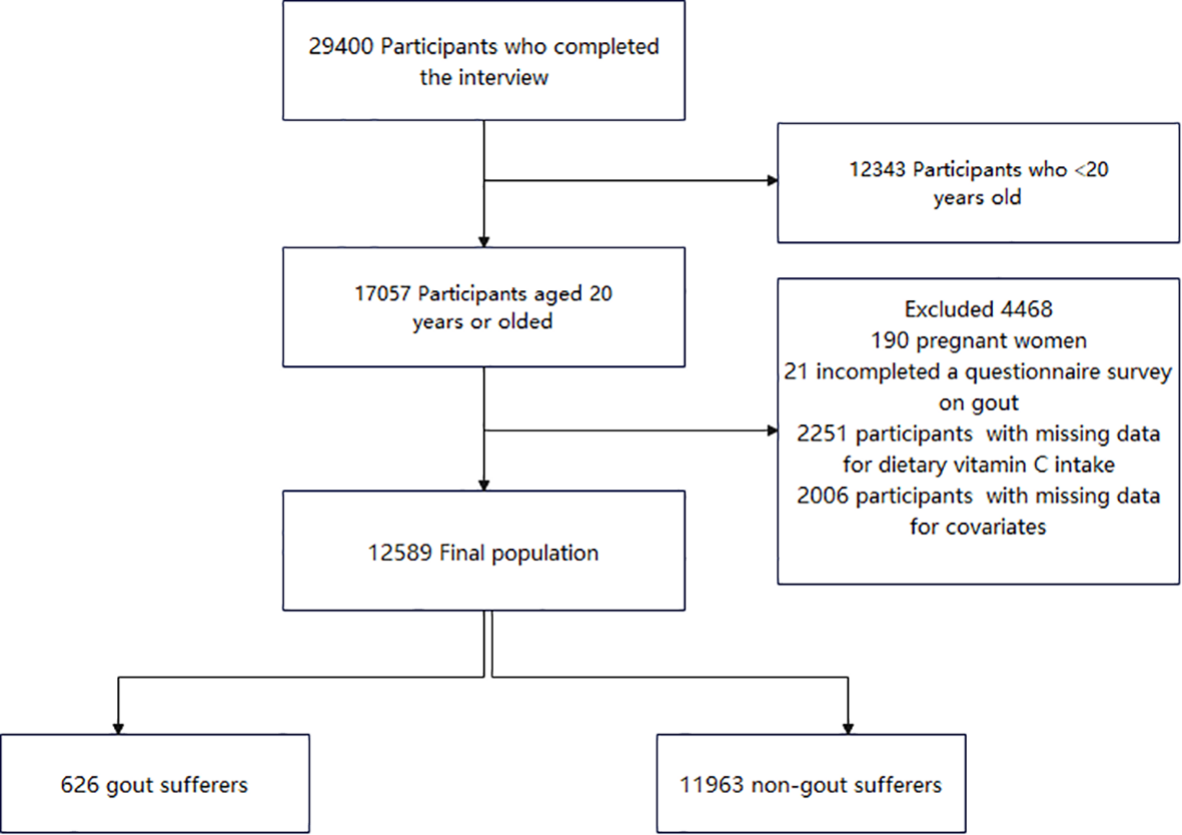
The study’s flow diagram.

In total, 29400 participants completed the interview, of whom 12343 participants were aged less than 20 years. We excluded pregnant females (n = 190), those with missing data on migraine (n = 652), those with missing data on dietary vitamin C intake (n = 2251), and those with covariates (n = 2006). Ultimately, this cross-sectional study included 12589 participants from the NHANES between 2013 and 2018. The detailed inclusion and exclusion criteria are presented in **Figure 1**.

**Table 1** presents the characteristics of 12,589 adults from NHANES between 2013 and 2018, categorized by dietary vitamin C intake quartiles: Q1 (≤19.9 mg/day), Q2 (19.9-49.7 mg/day), Q3 (49.7–110.375 mg/day), and Q4 (≥110.375 mg/day). The average age was 49.9 ± 17.5 years, with a significant difference across quartiles (P < 0.001). Age increased from 48.3 ± 17.3 years in Q1 to 50.3 ± 17.6 years in Q4. The sex distribution varied, with males comprising 48.9% of Q1 and 53% of Q4 (*P* < 0.001). Regarding marital status, 59.7% were married or living with a partner, with higher proportions in Q3 and Q4. Regarding race/ethnicity, the proportion of non-Hispanic whites decreased from 44.4% in Q1 to 35.5% in Q4, whereas the percentage of Mexican Americans increased from 12.1% to 15.3% (*P* < 0.001). Educational and family income levels were significantly higher in Q4 than in Q1 (*P* < 0.001). Health conditions such as hypertension showed no significant variation across quartiles, whereas diabetes prevalence was the lowest in Q4 (12.1%, *P* < 0.001). Smoking status and dietary supplement use were positively associated with higher vitamin C intake. Physical activity levels indicated a greater proportion of sedentary lifestyles in the higher quartiles (*P* < 0.001).

**Table 1:** Population characteristics by categories of dietary vitamin C intake.

Characteristic	Total Population (n = 12589)	Quartile 1 (n = 3177)	Quartile 2 (n = 3142)	Quartile 3 (n = 3148)	Quartile 4 (n = 3122)	*P*-value
Age, Mean ± SD	49.9 ± 17.5	48.3 ± 17.3	49.8 ± 17.4	51.3 ± 17.5	50.3 ± 17.6	< 0.001
Sex, n (%)						< 0.001
Male	6203 (49.3)	1552 (48.9)	1495 (47.6)	1502 (47.7)	1654 (53)	
Female	6386 (50.7)	1625 (51.1)	1647 (52.4)	1646 (52.3)	1468 (47)	
Marital status, n (%)						< 0.001
Married or living with a partner	7513 (59.7)	1742 (54.8)	1910 (60.8)	1948 (61.9)	1913 (61.3)	
Living alone	5076 (40.3)	1435 (45.2)	1232 (39.2)	1200 (38.1)	1209 (38.7)	
Race/ethnicity, n (%)						< 0.001
Non-Hispanic white	5062 (40.2)	1409 (44.4)	1301 (41.4)	1245 (39.5)	1107 (35.5)	
Non-Hispanic black	2639 (21.0)	743 (23.4)	625 (19.9)	619 (19.7)	652 (20.9)	
Mexican American	1784 (14.2)	386 (12.1)	455 (14.5)	465 (14.8)	478 (15.3)	
Others	3104 (24.7)	639 (20.1)	761 (24.2)	819 (26)	885 (28.3)	
Education level (year), n (%)						< 0.001
< 9	990 (7.9)	234 (7.4)	254 (8.1)	273 (8.7)	229 (7.3)	
9~12	4369 (34.7)	1360 (42.8)	1113 (35.4)	990 (31.4)	906 (29)	
>12	7230 (57.4)	1583 (49.8)	1775 (56.5)	1885 (59.9)	1987 (63.6)	
Family income, n (%)						< 0.001
Low	3896 (30.9)	1196 (37.6)	954 (30.4)	863 (27.4)	883 (28.3)	
Medium	4873 (38.7)	1258 (39.6)	1252 (39.8)	1216 (38.6)	1147 (36.7)	
High	3820 (30.3)	723 (22.8)	936 (29.8)	1069 (34)	1092 (35)	
Hypertension, n (%)	3823 (30.4)	968 (30.5)	937 (29.8)	987 (31.4)	931 (29.8)	0.504
DM, n (%)	1825 (14.5)	464 (14.6)	498 (15.8)	484 (15.4)	379 (12.1)	< 0.001
CHD, n (%)	556 (4.4)	134 (4.2)	144 (4.6)	135 (4.3)	143 (4.6)	0.844
Smoking status, n (%)						< 0.001
Never	7084 (56.3)	1563 (49.2)	1720 (54.7)	1862 (59.1)	1939 (62.1)	
Current	3078 (24.4)	689 (21.7)	807 (25.7)	831 (26.4)	751 (24.1)	
Former	2427 (19.3)	925 (29.1)	615 (19.6)	455 (14.5)	432 (13.8)	
Alcohol, n (%)	9754 (77.5)	2518 (79.3)	2442 (77.7)	2392 (76)	2402 (76.9)	0.015
Physical activity, n (%)						< 0.001
Sedentary	7070 (56.2)	1699 (53.5)	1746 (55.6)	1828 (58.1)	1797 (57.6)	
Moderate	2746 (21.8)	662 (20.8)	702 (22.3)	701 (22.3)	681 (21.8)	
Vigorous	2773 (22.0)	816 (25.7)	694 (22.1)	619 (19.7)	644 (20.6)	
Dietary supplements taken, n (%)	6726 (53.4)	1434 (45.1)	1611 (51.3)	1858 (59)	1823 (58.4)	< 0.001

DM diabetes; CHD, coronary heart disease.

### 3.2 Prevalence of gout by vitamin c intake quartiles

Participants were categorized into quartiles based on dietary vitamin C intake: Q1 (≤19.9 mg/day), Q2 (19.9–49.7 mg/day), Q3 (49.7–110.375 mg/day), and Q4 (≥110.375 mg/day). The prevalence of gout decreased across quartiles, with Q1 showing the highest percentage at 5.40% (169 participants) and Q4 showing the lowest at 4.60% (142 participants). The total number of participants was 3,177 in Q1, 3,142 in Q2, 3,148 in Q3, and 3,122 in Q4, indicating an inverse relationship between vitamin C intake and gout risk (**Table 2**).

**Table 2:** Gout Incidence and Vitamin C Intake by Quartiles.

Quartile	Vitamin C Intake (mg/day)	Participants with Gout	Total Participants	Percent with Gout
Quartile 1	≤19.9	169 (5.3)	3177	5.40%
Quartile 2	19.9-49.7	159 (5.1)	3142	4.90%
Quartile 3	49.7-110.375	156 (5)	3148	4.90%
Quartile 4	≥110.375	142 (4.5)	3122	4.60%

### 3.3 Relationship between dietary vitamin C intake and gout

**Table 1** presents the OR and 95% CI for gout-related factors. Age showed a significant association (OR = 1.05, 95% CI: 1.04–1.06, P < 0.001), indicating a higher risk with increasing age. Females had a lower risk than males (OR = 0.39, 95% CI: 0.33–0.46, P < 0.001). Living alone was associated with a reduced risk compared to being married or living with a partner (OR = 0.83, 95% CI: 0.7–0.98, *P* = 0.027). Among racial/ethnic groups, Mexican Americans showed a significantly lower risk (OR = 0.51, 95% CI: 0.37–0.69, *P* < 0.001). Hypertension, diabetes, and coronary heart disease were strongly associated with a higher risk of gout, with ORs of 4.11, 3.44, and 4.27, respectively (all *P* < 0.001). Current smokers had a higher risk (OR = 2.52, 95% CI: 2.11–3.01, *P* < 0.001), while alcohol use and dietary supplement intake were also positively associated with gout risk (*P* = 0.002 and *P* < 0.001, respectively) (**Table 3**).

**Table 3:** Association of covariates and gout risk.

Variable	OR (95%CI)	*P* value
Age (year)	1.05 (1.04~1.06)	<0.001
Sex		
Male		
Female	0.39 (0.33~0.46)	<0.001
Marital status, n (%)		
Married or living with a partner	1 (reference)	
Living alone	0.83 (0.7~0.98)	0.027
Race/ethnicity, n (%)		
Non-Hispanic white	1 (reference)	
Non-Hispanic black	1.1 (0.9~1.34)	0.372
Mexican American	0.51 (0.37~0.69)	<0.001
Others	0.81 (0.65~0.99)	0.044
Education level (years), n (%)		
<9	1 (reference)	
9–12	1.36 (0.96~1.91)	0.081
>12	1.21 (0.87~1.69)	0.267
Family income, n (%)		
Low	1 (reference)	
Medium	1.01 (0.83~1.22)	0.935
High	1.02 (0.83~1.25)	0.843
Hypertension, n (%)		
No	1 (reference)	
Yes	4.11 (3.48~4.85)	<0.001
Diabetes, n (%)		
No	1 (reference)	
Yes	3.44 (2.9~4.09)	<0.001
Coronary heart disease, n (%)		
No	1 (reference)	
Yes	4.27 (3.36~5.43)	<0.001
Smoking status, n (%)		
Never	1 (reference)	
Current	2.52 (2.11~3.01)	<0.001
Former	1.19 (0.95~1.51)	0.135
Alcohol, n (%)		
No	1 (reference)	
Yes	1.4 (1.13~1.73)	0.002
Physical activity, n (%)		
Sedentary	1 (reference)	
Moderate	1.1 (0.9~1.34)	0.336
Vigorous	0.92 (0.75~1.13)	0.441
Dietary supplements taken, n (%)	1.47 (1.24~1.73)	<0.001
Vitamin C consumption (per100mg/d)	0.91 (0.83~1.01)	0.07

**Figure 2:**
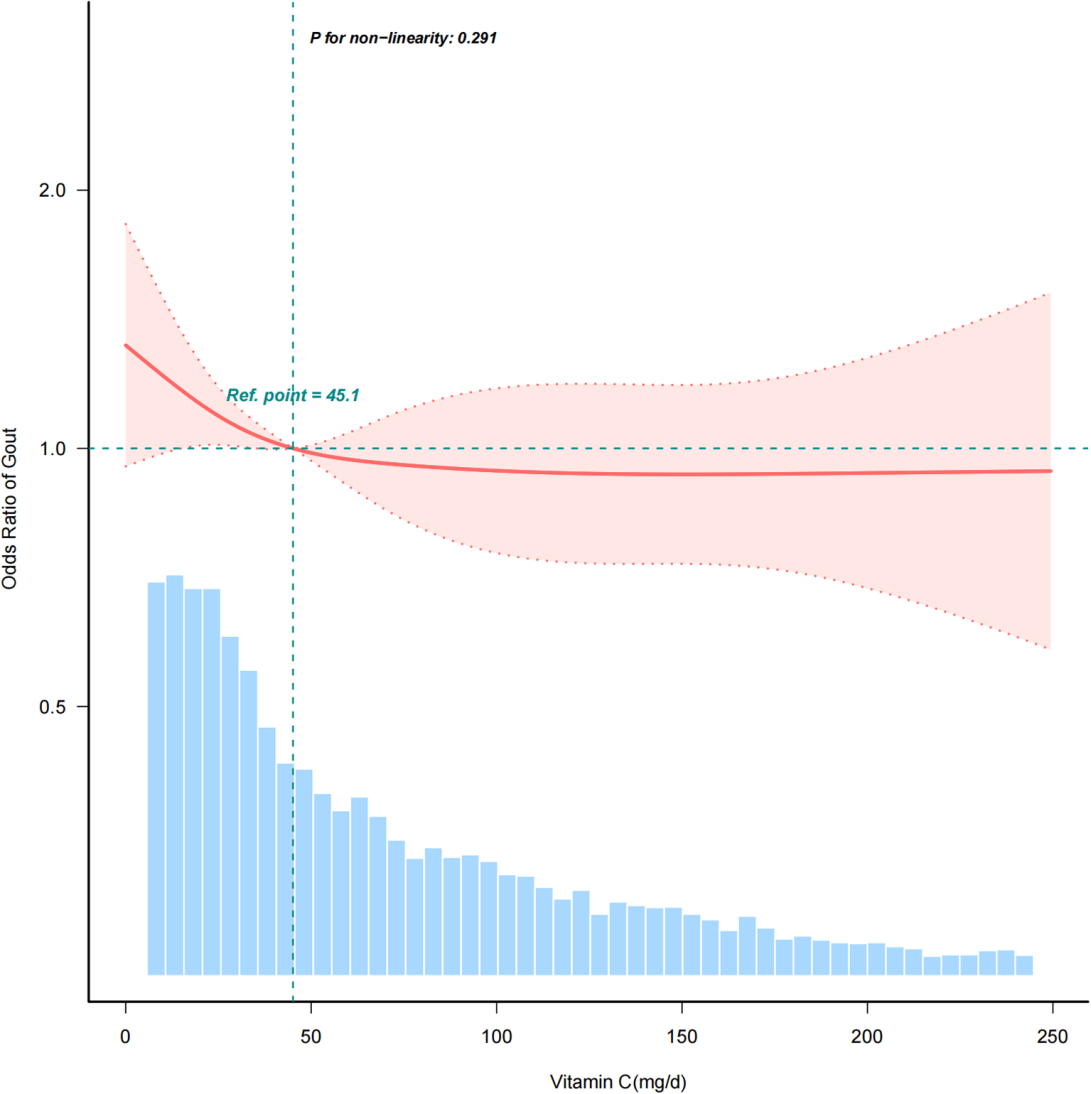
Association between dietary vitamin C intake and gout odds ratio. The solid and dashed lines represent the predicted values and 95% confidence intervals. Data were adjusted for sociodemographic factors (age, sex, marital status, race/ethnicity, education level, and family income), hypertension, diabetes, coronary heart disease, smoking status, physical activity, alcohol consumption, and dietary supplement intake.

**Table 4** illustrates that after adjusting for other risk factors, there is a significant correlation between vitamin C intake and gout (OR = 0.88, 95% CI: 0.79–0.97, *P* = 0.015). When dietary vitamin C consumption was analyzed using quartiles, there was a significant inverse association between dietary vitamin C and gout after adjusting for potential confounders. Compared with individuals with lower vitamin C consumption in Q1 group (≤19.9 mg/day), the adjusted OR values for dietary vitamin C intake and gout in the Q2 group (19.9-49.7 mg/day), the Q3 group (49.7-110.375 mg/day), and the Q4 group (≥110.375 mg/day) were 0.87 (95% CI: 0.69–1.1, *P* = 0.237), 0.81 (95% CI: 0.64–1.02, *P* = 0.076), and 0.77 (95% CI: 0.6–0.99, *P* = 0.042) (**Table 4**), respectively. The association between dietary vitamin C intake and gout exhibited an L-shaped curve (nonlinear, *P*= 0.245) in RCS (**Figure 2**).

**Table 4:** Association between dietary vitamin C (from the first survey) and gout.

Variable	N	Gout, n,%	Crude	*P* value	Model 1	OR (95%CI)	Model 2	*P* value	Model 3	*P* value
			OR (95%CI)		OR (95%CI)		*P* value		OR (95%CI)	
Vitamin C (per 100mg/d)	12589	626 (5)	0.91 (0.83~1.01)	0.07	0.86 (0.78~0.96)	0.007	0.87 (0.79~0.97)	0.012	0.88 (0.79~0.97)	0.015
Quartiles										
Quartile 1	3177	169 (5.3)	1(Ref)		1(Ref)		1(Ref)		1(Ref)	
Quartile 2	3142	159 (5.1)	0.95 (0.76~1.18)	0.643	0.89 (0.71~1.13)	0.341	0.89 (0.71~1.13)	0.334	0.89 (0.7~1.12)	0.322
Quartile 3	3148	156 (5)	0.93 (0.74~1.16)	0.512	0.82 (0.65~1.03)	0.091	0.81 (0.64~1.03)	0.086	0.81 (0.64~1.03)	0.082
Quartile 4	3122	142 (4.5)	0.85 (0.67~1.07)	0.158	0.73 (0.57~0.92)	0.009	0.74 (0.58~0.95)	0.016	0.75 (0.59~0.95)	0.019
*P* for trend	12589	626 (5)	0.95 (0.88~1.02)	0.163	0.9 (0.83~0.97)	0.006	0.91 (0.84~0.98)	0.012	0.91 (0.84~0.98)	0.014

OR, odds ratio; CI, confidence interval; Ref: reference. Model 1 was adjusted for sociodemographic variables (age, sex, marital status, race/ethnicity, education level, family income). Model 2 was adjusted for sociodemographic (age, sex, marital status, race/ethnicity, education level, and family income), hypertension, diabetes, coronary heart disease. Model 3 was adjusted for sociodemographic (age, sex, marital status, race/ethnicity, education level, and family income), hypertension, diabetes, coronary heart disease, smoking status, physical activity, alcohol, and dietary supplements taken.

### 3.4 Stratified analyses based on additional variables

**Figure 3:**
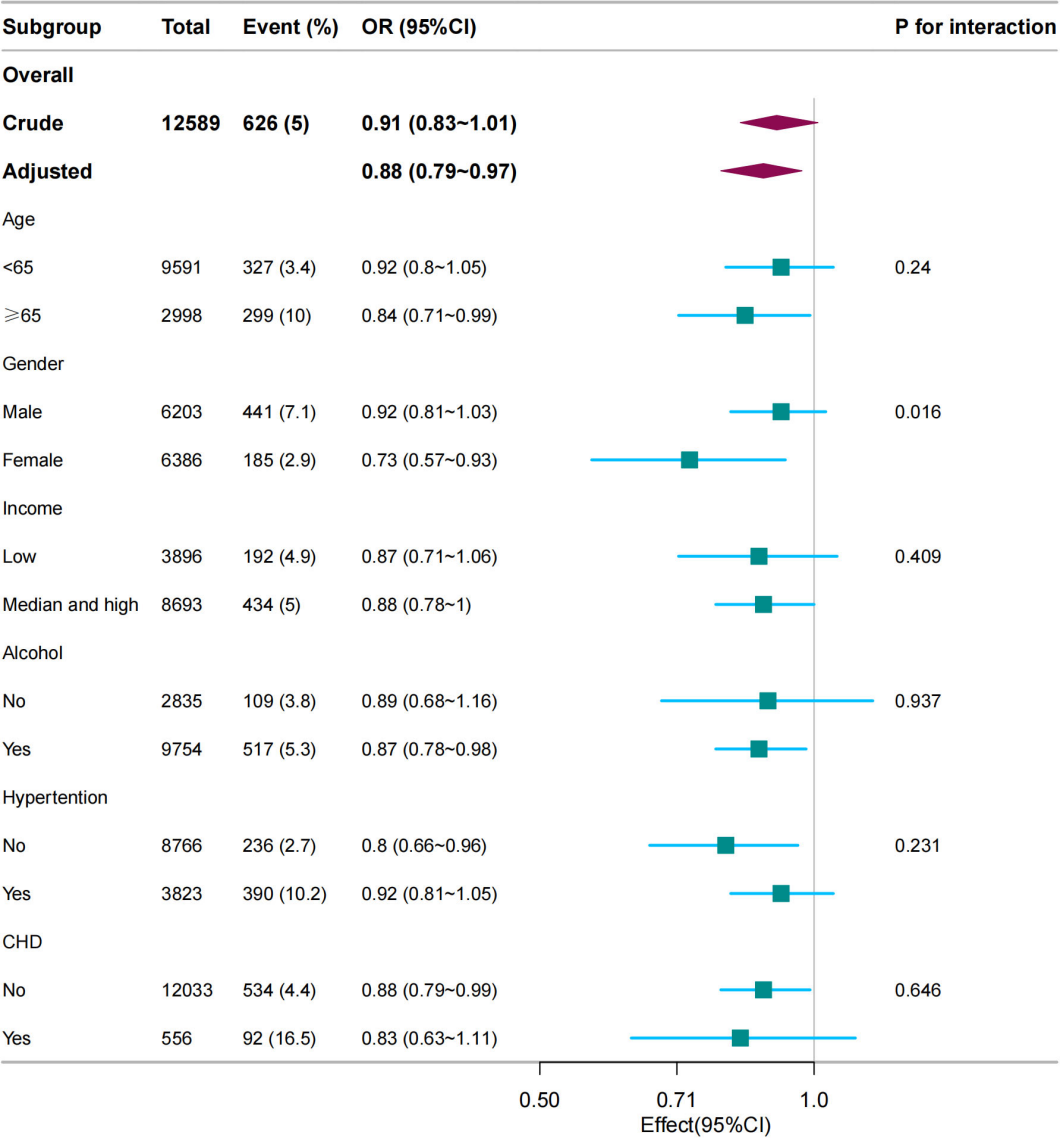
Subgroup analysis between dietary vitamin C (per 100 mg/day) and gout. Except for the stratification component, each stratification factor was adjusted for age, sex, marital status, race/ethnicity, educational level, family income, hypertension, diabetes, coronary heart disease, smoking status, physical activity, alcohol consumption, and dietary supplement intake.

In several subgroups, stratified analysis was performed to assess potential effect modifications in the relationship between dietary vitamin C and gout. Subgroup analysis revealed significant interactions between vitamin C levels and gout, notably influenced by sex, suggesting the need for tailored interventions for at-risk populations. No significant interactions were found in all subgroups after stratification by age, income, alcohol consumption, hypertension, and coronary heart disease (**Figure 3**). Considering multiple testing, a *P* value < 0.05 for the interaction of sex is statistically significant.

### 3.5 Sensitivity analyses

After excluding individuals with extreme vitamin C intake (≥1000 mg/day), 12584 individuals remained, and the association between vitamin C intake and gout remained stable (**Supplementary Table 1**). Compared with individuals with lower vitamin C consumption in Q1 group (≤19.9 mg/day), the adjusted OR values for dietary vitamin C intake and gout in Q4 group (≥110.375 mg/day) were 0.75 (95% CI: 0.59–0.95, *P* = 0.02).

In the univariate analysis, vitamin C (per 100mg/d) from the second survey is associated with gout (OR, 0.86; 95% CI 0.77–0.96, *P* = 0.01). After adjusting for all covariates, the association between vitamin C from the second survey and gout remained (OR, 0.81; 95%CI, 0.72–0.91, *P* < 0.001).

Compared with individuals with lower vitamin C consumption Q1 (≤19.9 mg/day), the adjusted OR values for dietary vitamin C from the second survey and gout in Q2 (19.9–49.7 mg/day), Q3 (49.7–110.375 mg/day), and Q4 (≥110.375 mg/day) were 0.95(95% CI, 0.74–1.21, *P* = 0.65), 0.73 (95% CI, 0.57–0.94, *P* = 0.014), and 0.64 (95% CI, 0.49–0.84, *P* = 0.001) (**Supplementary Table 2**), respectively.

The adjusted OR between dietary supplementation with vitamin C (per 100mg/d) and gout was 0.997 (95% CI, 0.964–1.031; *P* = 0.8602) (**Supplementary Table 3**).

Vitamin C was also negatively associated with hyperuricemia (OR, 0.94; 95% CI, 0.9–0.98, *P* = 0.005). Compared with Q1 group (≤19.9 mg/day), the adjusted OR values for dietary vitamin C from the first survey and hyperuricemia in Q2 group (19.9–49.7 mg/day), Q3 group (49.7–110.375 mg/day), and Q4 group (≥110.375 mg/day) were 0.77 (95% CI, 0.69–0.86, *P* = 0.65), 0.81 (95% CI, 0.72–0.91, *P* = 0.014), and 0.72 (95% CI, 0.64–0.81, *P* < 0.001) (**Supplementary Table 4**), respectively.

### 3.6 Model comparison and robustness

**Supplementary Table 5** shows that, while Model 3 had a slightly higher BIC than Model 2, it performed the best in terms of AIC and log-likelihood, suggesting that Model 3 may be more suitable for our data.

## 4 Discussion

This large cross-sectional study on American adults demonstrated a significant inverse relationship between dietary vitamin C consumption and gout. Stratified analyses showed that the relationship between vitamin C consumption and gout remained robust. The inverse correlations between dietary vitamin C and gout were more significant in females than in males. Vitamin C levels are also negatively associated with hyperuricemia. However, dietary supplemental vitamin C was not associated with gout.

The influence of vitamin C’s influence on gout has been documented in only a few cases. Our results showed that participants with a higher intake of vitamin C exhibited a reduced risk of developing gout, which aligns with the findings of a previous study (18). The physicians’ Health Study II also suggested that 500 mg/day of vitamin C modestly reduced the risk of new gout diagnoses in middle-aged male physicians (19). However, our study extends these findings to a broader demographic group, including various ethnic groups, income groups, and both sexes.

MW et al. (6) demonstrated that the highest quartile of dietary VC intake was negatively associated with the risk of hyperuricemia in males. The potential of vitamin C to reduce the risk of hyperuricemia has also been suggested in a population-based study, in which greater vitamin C intake was associated with a lower prevalence of hyperuricemia (14) (SUA > 6 mg/dL). However, our study provides evidence from a large population that vitamin C supplementation may reduce the incidence of gout. Nevertheless, our study did not find evidence to support the efficacy of vitamin C beyond current pharmacological treatments. Therefore, it is important to emphasize that our results should not be interpreted as a reason to neglect urate-lowering therapies in adults who have previously experienced gout.

Despite consistency with previous research, our findings diverged from those reported by Stamp (20), who found no significant association between vitamin C intake and SUA levels in a pilot randomized controlled trial. This discrepancy could be attributed to differences in study design, population characteristics, or the range of vitamin C intake. Unlike the wide range of vitamin C levels in our study, Stamp et al. studied only the effect of vitamin C at a dose of 500 mg/day on uric acid levels. Moreover, Stamp recruited only 40 patients, possibly leading to different statistical biases.

Our study observed no clear association between supplemental vitamin C and gout. Previous intervention studies have also found that supplemental VC does not influence SUA levels (20, 21). First, the bioavailability of vitamin C in supplements may differ from that in natural food sources. Vitamin C in whole foods is often accompanied by other nutrients, such as bioflavonoids, which may enhance its antioxidant and anti-inflammatory effects, potentially making it more effective in preventing gout (22). Second, genetic variations in the pathways responsible for maintaining redox balance and vitamin C transportation correlate with serum vitamin C concentrations (23). Serum vitamin C levels tend to plateau at an intake threshold of approximately 200 mg/day; beyond this point, serum vitamin C concentrations do not increase with higher supplemental doses (23, 24). In essence, a combination of genetic factors and dietary intake dictate serum vitamin C levels, and additional vitamin C supplementation may not negatively correlate with SUA levels when the serum vitamin C levels are already elevated.

The findings revealed that the inverse correlation between dietary vitamin C intake and gout was more significant in females than in males. These results can be interpreted from several perspectives. First, there were notable differences in metabolism and hormone levels between females and males. Estrogen inhibits uric acid production and promotes its excretion, which may enhance the protective effects of higher vitamin C intake in females (25). Second, differences in dietary habits and lifestyles between sexes may also contribute to the observed results. For example, females may be more likely to maintain a balanced diet with adequate vitamin C intake, which could lead to a more pronounced effect of vitamin C in reducing gout risk (26). Third, female participants were more likely to engage in healthier lifestyle practices or meet the recommended nutritional standards, which might exaggerate the inverse relationship between vitamin C and gout (27). These factors need to be accounted for when interpreting the results as they could introduce bias into the findings.

One of the primary limitations of this study is its cross-sectional design, which inhibited the ability to establish causality between vitamin C intake and gout incidence. Longitudinal studies are needed to confirm these findings and better understand the temporal relationships. Additionally, while the NHANES provides a nationally representative sample of the U.S. population, the generalizability of these findings to other populations may be limited owing to cultural and dietary differences. Another limitation concerns potential residual confounding, as not all factors influencing the risk of gout, such as genetic predispositions or detailed dietary components, were fully accounted for. Fourth, dietary vitamin C intake was assessed via a 24-h recall, potentially introducing recall bias. Nevertheless, the food frequency survey offers less detailed information on food types and quantities than the 24-h recall (28, 29).

In conclusion, there is a negative association between dietary vitamin C intake and gout prevalence among adults in the United States. The results of this study highlight the association between dietary vitamin C intake and gout.

## Data Availability

The original contributions presented in the study are included in the article/Supplementary Material. Further inquiries can be directed to the corresponding author.
