# Supplementary material for: Association between dietary vitamin C intake and gout among American adults
Source: Front Immunol. 2024 Sep 13;15:1431323. doi: 10.3389/fimmu.2024.1431323 (PMC11437527; doi:10.3389/fimmu.2024.1431323)
Supplement: Supplementary file 2 [file DataSheet2.pdf]

frontiers

Supplementary Material

1 Supplementary Figures and Tables

1.1 Supplementary Tables

Supplementary Table 1. Association between dietary vitamin C (<1000 mg/day) intake and gout

		Vitamin C intake(mg)				
	Variable	Quartile 1	Quartile 2	Quartile 3	Quartile 4	*P* for trend
	N	3146	3146	3146	3146	12584
	Gout,%	167 (5.3)	160 (5.1)	156 (5)	143 (4.5)	626 (5)
Model1	OR (95%CI)	1(Ref)	0.9 (0.72~1.13)	0.81 (0.64~1.03)	0.72 (0.57~0.92)	-
	P value		0.375	0.082	0.008*	0.005*
Model2	OR (95%CI)	1(Ref)	0.9 (0.71~1.13)	0.82 (0.64~1.03)	0.74 (0.58~0.95)	-
	P value		0.364	0.091	0.016*	0.011*
Model3	OR (95%CI)	1(Ref)	0.9 (0.71~1.13)	0.81 (0.64~1.03)	0.75 (0.59~0.95)	-

P value	0.358	0.09	0.02*	0.014*

OR, odds ratio; CI, confidence interval; Ref: reference.

Model 1 was adjusted for sociodemographic variables (age, sex, marital status, race/ethnicity, education level, family income).

Model 2 was adjusted for sociodemographic (age, sex, marital status, race/ethnicity, education level, family income), hypertension, diabetes, coronary heart disease.

Model 3 was adjusted for sociodemographic (age, sex, marital status, race/ethnicity, education level, family income), hypertension, diabetes, coronary heart disease, smoking status, physical activity, alcohol, dietary supplements taken. $P<0.05$, *

Supplementary Table 2. Association between dietary vitamin C intake (from the second survey) and gout

			Crude		Model1		Model 2		Model3	
Variables	N	Gout, n,%	OR (95%CI)	*P* value	OR (95%CI)	*P* value	OR (95%CI)	*P* value	OR (95%CI)	*P* value
Vitamin C (per 100 mg/d)	11030	566 (5.1)	0.86(0.77~0.96)	0.01	0.80 (0.71~0.90)	<0.001*	0.81(0.72~0.91)	<0.001*	0.81(0.72~0.91)	<0.001*
Quartiles										
Quartile 1	2756	151 (5.5)	1(Ref)		1(Ref)		1(Ref)		1(Ref)	
Quartile 2	2751	161 (5.9)	1.07 (0.85~1.35)	0.549	0.95 (0.74~1.2)	0.645	0.95 (0.74~1.21)	0.671	0.95 (0.74~1.21)	0.65
Quartile 3	2763	137 (5)	0.9 (0.71~1.14)	0.385	0.73 (0.57~0.94)	0.015*	0.73 (0.57~0.94)	0.014*	0.73 (0.57~0.94)	0.014*
Quartile 4	2760	117 (4.2)	0.76 (0.6~0.98)	0.033*	0.62 (0.48~0.8)	<0.001*	0.65 (0.5~0.84)	0.001*	0.64 (0.49~0.84)	0.001*
P for trend	11030	566 (5.1)	-	0.014*	-	<0.001*	-	<0.001*	-	<0.001*

OR, odds ratio; CI, confidence interval; Ref: reference.

Model 1 was adjusted for sociodemographic variables (age, sex, marital status, race/ethnicity, education level, family income).

Model 2 was adjusted for sociodemographic (age, sex, marital status, race/ethnicity, education level, family income), hypertension, diabetes, coronary heart disease.

Model 3 was adjusted for sociodemographic (age, sex, marital status, race/ethnicity, education level, family income), hypertension, diabetes, coronary heart disease, smoking status, physical activity, alcohol, dietary supplements taken.

P<0.05, *

Supplementary Table 3. Association between dietary supplemental vitamin C and gout

Variable	N	Gout, %	Crude		Model1		Model2		Model3	
			OR (95%CI)	*P* value	OR (95%CI)	*P* value	OR (95%CI)	*P* value	OR (95%CI)	*P* value
Vitamin C (per100 mg/d)	3177	192 (6)	0.998 (0.967~1.03)	0.9126	0.989 (0.955~1.025)	0.552	0.998 (0.964~1.032)	0.8919	0.997 (0.964~1.031)	0.8602
Quartiles										
Quartile 1	343	15 (4.4)	1(Ref)		1(Ref)		1(Ref)		1(Ref)	
Quartile 2	1141	77 (6.7)	1.58 (0.9~2.79)	0.112	1.00 (0.56~1.82)	0.963	0.99 (0.54~1.79)	0.965	1.00 (0.55~1.84)	0.981
Quartile 3	880	50 (5.7)	1.32 (0.73~2.38)	0.361	0.9 (0.49~1.65)	0.73	0.92 (0.49~1.71)	0.791	0.94 (0.5~1.76)	0.845

Quartile 4	813	50 (6.2)	1.43 (0.79~2.59)	0.233	0.9 (0.49~1.65)	0.728	0.96 (0.52~1.79)	0.896	0.98 (0.53~1.83)	0.953
P for trend	317 7	192 (6)	1.03 (0.88~1.19)	0.743	0.95 (0.81~1.11)	0.501	0.98 (0.83~1.16)	0.822	0.99 (0.84~1.16)	0.859

OR, odds ratio; CI, confidence interval; Ref: reference.

Model 1 was adjusted for sociodemographic variables (age, sex, marital status, race/ethnicity, education level, family income).

Model 2 was adjusted for sociodemographic (age, sex, marital status, race/ethnicity, education level, and family income), hypertension, diabetes, coronary heart disease.

Model 3 was adjusted for sociodemographic (age, sex, marital status, race/ethnicity, education level, family income), hypertension, diabetes, coronary heart disease, smoking status, physical activity, alcohol, dietary supplements taken. *P*<0.05, *

Supplementary Table 4. Association between Vitamin C intake and hyperuricemia

			Crude		Model 1		Model 2		Model 3	
Variables	N	Hyperuricemi a,%	OR (95%CI)	*P* value	OR (95%CI)	*P* value	OR (95%CI)	*P* value	OR (95%CI)	*P* value
Vitamin C (per 100mg/d)	1201 9	4097 (34.1)	0.99 (0.95~1.03)	0.725	0.94 (0.9~0.98)	0.007*	0.94 (0.9~0.98)	0.005*	0.94 (0.9~0.98)	0.005*
Quartiles										

Quartile 1	3018	1126 (37.3)	1(Ref)		1(Ref)		1(Ref)		1(Ref)	
Quartile 2	3029	977 (32.3)	0.8 (0.72~0.89)	<0.001*	0.78 (0.7~0.88)	<0.001*	0.78 (0.7~0.88)	<0.001*	0.77 (0.69~0.86)	<0.001*
Quartile 3	2985	1004 (33.6)	0.85 (0.77~0.95)	0.003*	0.83 (0.74~0.93)	0.001*	0.83 (0.74~0.93)	0.002*	0.81 (0.72~0.91)	<0.001*
Quartile 4	2987	990 (33.1)	0.83 (0.75~0.93)	0.001*	0.74 (0.66~0.83)	<0.001*	0.74 (0.66~0.83)	<0.001*	0.72 (0.64~0.81)	<0.001*
P for trend	12019	4097 (34.1)	-	0.004*	-	<0.001*	-	<0.001*	-	<0.001*

OR, odds ratio; CI, confidence interval; Ref: reference.

Model 1 was adjusted for sociodemographic variables (age, sex, marital status, race/ethnicity, education level, family income).

Model 2 was adjusted for sociodemographic (age, sex, marital status, race/ethnicity, education level, and family income), hypertension, diabetes, coronary heart disease.

Model 3 was adjusted for sociodemographic (age, sex, marital status, race/ethnicity, education level, and family income), hypertension, diabetes, coronary heart disease, smoking status, physical activity, alcohol, dietary supplements taken.

Supplementary Table 5. Likelihood ratios for the regression

	Model 1	Model2	Model 3
Number of Observations	12589	12589	12589

AIC	4459.4	4308	4302.3
BIC	4548.7	4419.6	4458.6
Log.Lik.	-2217.7	-2139	-2130.2
RMSE	0.21	0.21	0.21

AIC, Akaike's Information Criterion; BIC, Bayesian Information Criterion (*BIC*) Log.lik, Log Likelihood. RMSE, Root mean square error.;

Model 1 was adjusted for sociodemographic variables (age, sex, marital status, race/ethnicity, education level, family income).

Model 2 was adjusted for sociodemographic (age, sex, marital status, race/ethnicity, education level, family income), hypertension, diabetes, coronary heart disease.

Model 3 was adjusted for sociodemographic (age, sex, marital status, race/ethnicity, education level, family income), hypertension, diabetes, coronary heart disease, smoking status, physical activity, alcohol, dietary supplements taken.
